# Fast-food-based hyper-alimentation can induce rapid and profound elevation of serum alanine aminotransferase in healthy subjects

**DOI:** 10.1136/gut.2007.131797

**Published:** 2008-02-14

**Authors:** S Kechagias, Å Ernersson, O Dahlqvist, P Lundberg, T Lindström, F H Nystrom, for the Fast Food Study Group

**Affiliations:** 1 Division of Internal Medicine, Department of Medicine and Care, Faculty of Health Sciences, Linköping University, Linköping, Sweden; 2 Division of Radiation Physics and Radiology, Center for Medical Image Science and Visualization, Department of Medicine and Care, Faculty of Health Sciences, Linköping University, Linköping, Sweden; 3 Diabetes Research Centre. Linköping University, Linköping, Sweden

## Abstract

**Objective:**

To study the effect of fast-food-based hyper-alimentation on liver enzymes and hepatic triglyceride content (HTGC).

**Design:**

Prospective interventional study with parallel control group.

**Setting:**

University Hospital of Linköping, Sweden.

**Participants:**

12 healthy men and six healthy women with a mean (SD) age of 26 (6.6) years and a matched control group.

**Intervention:**

Subjects in the intervention group aimed for a body weight increase of 5–15% by eating at least two fast-food-based meals a day with the goal to double the regular caloric intake in combination with adoption of a sedentary lifestyle for 4 weeks.

**Main outcome measures:**

Weekly changes of serum aminotransferases and HTGC measured by proton nuclear magnetic resonance spectroscopy at baseline and after the intervention.

**Results:**

Subjects in the intervention group increased from 67.6 (9.1) kg to 74.0 (11) kg in weight (p<0.001). Serum ALT increased from 22.1 (11.4) U/l at study start to an individual mean maximum level of 97 (103) U/l (range 19.4–447 U/l). Eleven of the 18 subjects persistently showed ALT above reference limits (women >19 U/l, men >30 U/l) during the intervention. Sugar (mono- and disaccharides) intake during week 3 correlated with the maximal ALT/baseline ALT ratio (r = 0.62, p = 0.006). HTGC increased from 1.1 (1.9)% to 2.8 (4.8)%, although this was not related to the increase in ALT levels. ALT levels were unchanged in controls.

**Conclusion:**

Hyper-alimentation *per se* can induce profound ALT elevations in less than 4 weeks. Our study clearly shows that in the evaluation of subjects with elevated ALT the medical history should include not only questions about alcohol intake but also explore whether recent excessive food intake has occurred.

Liver disease is often identified by asymptomatic elevations in serum aminotransferases, as these are commonly included in serum chemistry panels drawn on healthy individuals. However, incidentally discovered elevations of serum aminotransferases often lead to lengthy, expensive and potentially invasive diagnostic evaluations. Analysis of data from the Third National Health and Nutrition Examination Survey (NHANES III) in the United States showed a prevalence of elevated alanine aminotransferase (ALT) or aspartate aminotransferase (AST) to be as high as 7.9%.^1^ This figure is considerably higher than the 1–4% prevalence rate reported in earlier studies,^2^ ^3^ and indicates that the prevalence of abnormal liver tests in the general population has increased. Although many cases of elevated aminotransferase levels can be explained by the consumption of large quantities of alcohol or the presence of hepatitis C virus (HCV) infection, a great number of cases typically remain unexplained.^1^ In both men and women, elevations of aminotransferases are strongly associated with features of the metabolic syndrome, such as abdominal adiposity, high plasma glucose levels and dyslipidaemia,^4^^–^7 Indeed, high serum levels of AST and ALT are associated with future risk of developing type 2 diabetes.^8^ Most cases of aminotransferase elevations in seemingly healthy subjects have hitherto generally been attributed to non-alcoholic fatty liver disease (NAFLD),^1^ ^9^ and liver biopsy studies of patients referred for aminotransferase elevations have demonstrated liver steatosis as the most common histological finding.^10^^–^12 Interestingly, it was recently shown that subjects with NAFLD and elevated aminotransferases have a significant risk of developing end-stage liver disease and a lower chance of survival mainly because of cardiovascular disease.^13^ Accordingly, the widespread misconception that NAFLD is a mild disease with good prognosis has been highlighted^14^ and it is likely that physicians will be more concerned when facing patients with elevated aminotransferases. However, in most asymptomatic individuals with high aminotransferase levels, the elevation is intermittent and normal levels are found when testing is repeated within months.^15^ ^16^

Although accumulation of triglycerides within hepatocytes in NAFLD is a reversible process, it is unlikely that steatosis is reversed rapidly without any specific interventions and thus can explain non-persistent aminotransferase elevations. Moreover, 79% of subjects with hepatic steatosis have normal ALT levels^17^ suggesting that conditions other than fatty infiltration of the liver underlie many cases of the asymptomatic elevations of aminotransferases that are commonly found in the general population.

We performed a study of the effects of 4 weeks of fast-food-based hyper-alimentation on levels of serum ALT and on hepatic triglyceride content (HTGC) measured by proton nuclear magnetic resonance spectroscopy (^1^H-MRS). The aim of the study was to investigate the potential link between changes of serum ALT to the amount of hepatic fatty infiltration in healthy non-obese subjects during a positive energy balance resulting in a weight gain of 5–15%.

## METHODS

### Intervention group

By local advertising we recruited 12 men and six women as volunteers for the intervention arm of the study. All subjects except one were students, the majority of whom were medical students. All participants had to be willing to accept an increase in body weight of 5–15% and to eat at least two fast-food-based meals a day, preferably at well known fast-food restaurants. The cost for the food was consecutively reimbursed based on the food receipts. Physical activity was not to exceed 5000 steps per day. If a study subject reached a weight gain of 15% he or she terminated the study as soon as possible by re-performing the same study investigations as were done at baseline. The participants were free from current diseases as judged by medical check-up and history. Hepatitis B surface antigen (HBsAg) and HCV antibodies were not detectable in any subject. One of the participants had many years ago been diagnosed with coeliac disease and underwent a duodenal biopsy at the end of the study. This biopsy showed normal duodenal histology.

All subjects in the intervention group continually had contact with dieticians, by weekly meetings or by phone, during the study. The dietary advice was individually adjusted to result in an intake corresponding to doubling the present caloric requirement. If the subject was not able or willing to ingest the hamburger-based diet at any stage, it was changed to whatever food the participant accepted with the highest priority to achieve the calculated caloric intake and also, if the study subject still found it acceptable, a diet rich in protein and saturated animal fat. Habitual weekly alcohol consumption was assessed at study entry and all subjects were asked to keep alcohol intake unchanged during the study period. One of the participants was an abstainer and one subject consumed 340 g/week. The remaining 16 subjects reported an alcohol consumption of <140 g/week, which is the cut-off limit most often used to set the diagnosis of NAFLD.^18^

The composition of the diet was determined based on reports from 3 days before the study and another two 3-day periods at the end of the first or third weeks (or a week earlier in the one subject who ended the trial after 2 weeks). Food items that were bought in supermarkets to be consumed at less precise time points, such as butter and other foodstuffs to mainly be eaten at home, were averaged for the whole study period. In most cases the exact food composition given by the corresponding fast-food restaurant could be used as the source of information, but if such information was incomplete, food composition charts were used instead. The total caloric intake was also determined for the whole study period, and was based on receipts and interviews.

Blood for laboratory tests was drawn in the fasting state at baseline, i.e. before starting on the extra caloric intake, after 2 weeks on the fast-food-based diet, and at the end of the study, i.e. either at the end of fourth week or earlier if prematurely terminated. Blood was also drawn in the non-fasting state at the end of the first and the third study weeks, to monitor changes in serum liver enzyme levels, and at long-term follow-up after 6 months (in the fasting state) after the intervention, analysed as described elsewhere.^19^

Subjects of the intervention group underwent ^1^H-MRS of HTGC^20^ by using a Philips Achieva 1.5 T magnetic resonance system (Philips Medical Systems, Best, The Netherlands) with a single detection element of a four element SENSE body coil. Localiser imaging for prescription of ^1^H-MRS from two different volumes of interest (VOIs) were acquired using a balanced, fast field echo pulse sequence. The ^1^H-MRS acquisition parameters were: PRESS volume selection 20×20×20 mm^3^ VOI, repetition time  =  3 s, echo time (TE)  =  35 ms, dummy excitations  =  2 and averages  =  8). All spectral and imaging acquisitions including preparation phases were performed during single breath holds. Additional spectra using a TE of 50 ms were acquired in a limited number of examinations for determination of a correction factor for T_2_ relaxation of the lipid signal. A correction factor for the T_2_ relaxation time of the liver water signal was measured using an axially prescribed single slice multi-echo turbo spin echo pulse sequence, (TE  =  10, 20, 30, 40, 50 and 60 ms). Zero- and first-order phase corrections, a 10 Hz line broadening, and a least squares optimised spline baseline correction were applied. The water and lipid resonances were integrated from 6.0 to 3.3 ppm and 2.7 to 0.4 ppm, respectively. The lipid volume fractions (HTGC) were calculated as previously described.^20^ The upper limit of normal for HTGC was based on the results by Szczepaniak *et al*^21^ who examined the distribution of HTGC with ^1^H-MRS in 345 subjects with a low risk for hepatic steatosis, i.e. BMI <25 kg/m^2^, no glucose intolerance or excessive alcohol consumption and normal serum ALT.^22^ Hepatic steatosis was defined as an HTGC greater than 5.6%, which corresponded to the 95^th^ percentile in this low-risk group.

Basal metabolic rate was measured using a ventilated hood technique (Delta Trac, SensorMedics, Yorba Linda, CA, USA)^23^ in the fasting state in the morning and the mean value of one measurement per minute during the last 6 min of a 15 min period was calculated. The subjects were also subjected to dual energy *x* ray absorptiometry (Hologic 4500, Hologic, Waltham, MA, USA), for analysis of body composition. A biopsy of subcutaneous abdominal fat was done under local anaesthesia and non-invasive measurements of the vascular function were recorded, the results of which will be reported separately. All the investigations were performed at baseline and during the last week of the study, except for the recording of basal metabolic rate, which was also measured in the fasting state after 2 weeks on the diet. All anthropometric measurements were done by two research nurses.

### Control group

An age- and gender-matched control group was also recruited mainly to allow discrimination of changes of ALT induced by food intake from random fluctuations. The control group performed the laboratory investigations and anthropometric measurements at baseline and after 4 weeks, as well as measurement of basal metabolic rate.

### Statistics

Statistical calculations were done with SPSS 14.0 software (SPSS Inc. Chicago, IL, USA). Linear correlations were calculated, except as stated in the text. Comparisons within and between groups were done with the Student paired and unpaired two-tailed t test or as stated in the results section. Mean (SD) is given unless otherwise stated. Statistical significance was considered at the 5% level (p⩽0.05).

### Ethics

The study was approved by the Ethics Committee of Linköping University and performed in accordance with the Declaration of Helsinki. Written informed consent was obtained from all participating subjects.

## RESULTS

Seventeen of the 18 participants met the goal of a 5–15% increase in body weight by the intervention based on hyper-alimentation combined with a sedentary life style. Table 1 shows baseline anthropometric and laboratory data of all the participants, and the effects of the intervention.

**Table 1:** Anthropometric and laboratory data before and after the hyper-alimentation in the intervention group and before and after 4 weeks on regular diet and exercise habits in the control group

Variable	Interv. gp before diet	Interv. gp after diet	p Value for baseline vs after diet in interv. gp	Controls at baseline	p Value for baseline in controls vs interv. gp	Controls after 4 weeks	p Value for levels at study end in controls vs interv. gp
Age (years)	27 (6.6)			25 (3.5)	0.3		
Sex (M/F)	12/6			12/6			
Weight (kg)	67.6 (9.1)	74.0 (11)	<0.001	69.7 (8.4)	0.5	69.7 (8.7)	0.2
Body mass index (kg/m^2^)	21.9 (1.9)	23.9 (2.2)	<0.001	22.2 (2.1)	0.7	22.2 (2.2)	0.02
Sagittal abdominal diameter (cm)	18.4 (1.7)	20.4 (1.6)	<0.001	17.8 (1.3)	0.3	17.8 (1.4)	<0.0001
Waist circumference (cm)	76.4 (6.4)	83.1 (7.9)	<0.001	75.5 (5.8)	0.7	75.4 (6.0)	0.002
Hip circumference (cm)	86.5 (7.1)	90.4 (8.5)	0.03	89.0 (6.9)	0.3	89.8 (6.1)	0.8
HOMA	0.89 (0.42)	1.6 (0.83)	0.002	1.2 (0.86)	0.2	1.1 (0.53)	0.02
ALT (U/l)	22.1 (11)	69.3 (76)	0.01	20.2 (3.9)	0.7	22 (8.0)	0.001
AST (U/l)	28.1 (12)	39.6 (23)	0.07	25.5 (5.8)	0.8	26.3 (5.6)	0.02
ALP (U/l)	57.3 (30)	61.2 (25)	0.1	52.7 (12)	0.6	52 (14)	0.2
Bilirubin (mg/dl)	0.70 (0.20)	0.55 (0.15)	0.01	0.89 (0.37)	0.1	0.75 (0.26)	0.008
Albumin (g/dl)	4.1 (0.34)	4.0 (0.27)	0.2	4.4 (0.35)	0.01	4.3 (0.33)	0.002
Hepatic triglyceride content (%)	1.1 (1.9)	2.8 (4.8)	0.003*	ND		ND	
Body fat (%)	20.1 (9.8)	23.8 (8.6)	<0.001	ND		ND	
Basal metabolic rate (kcal/24 h)	1615 (276)	1813 (327)	0.001	1700 (243)	0.3	1712 (262)	0.3

Figures are means (SD).

Interv. gp., intervention group.

*Comparison made with the Wilcoxon signed rank test. Conversions: ALT, AST and ALP (U/l) ×0.017 = μkat/l, bilirubin (mg/dl) ×17.1 = μmol/l, albumin (g/dl) ×10 = g/l. BMI, body mass index; HOMA, homeostasis model assessment;^24^ ALT, alanine aminotransferase; AST, aspartate aminotransferase; ALP, alkaline phosphatase, ND; not determined.

The mean daily caloric intake of the whole intervention period increased +70 (35)% (men +68 (31)%, women +74 (45)%, p = 0.8 for comparison of genders). Two men and two women had a mean daily caloric intake >+90% of basal during the entire study. There was no statistically significant change in the food intake of macronutrients or fibre when comparing the registration in the first and third weeks nor were there any gender differences with regard to relative intake of macronutrients. Table 2 displays food intake and the relative composition of the food before and during the study in the intervention group.

**Table 2:** Food composition before and during the end of the first week of hyper-alimentation in the intervention group

	Energy from fat, carbohydrates and protein (kcal/day)	Energy from fat (kcal/day)	Energy from fat (%)	Energy from carbohydrates (kcal/day)	Energy from carbohydrates (%)	Energy from protein (kcal/day)	Energy from protein (%)	Fat (g/day)	Sat. fat (g/day)	Monounsat. fat (g/day)	Polyunsat. fat (g/day)	Sugar (g/day)	Fibre (g/day)
Before study	2273 (558)	817 (240)	36 (5.7)	1099 (297)	48 (5.4)	357 (84)	16 (1.8)	87 (25)	33 (11)	32 (10)	16 (6.4)	95 (42)	26 (6)
During study	5753 (1495)	2457 (728)	43 (6.8)	2575 (743)	45 (7.2)	721 (249)	12 (2.2)	261 (77)	111 (36)	96 (30)	30 (10)	285 (117)	37 (15)

Figures are means (SD). All changes compared to baseline were statistically significant except those of the energy% from carbohydrates and the intake of fibre. Sugar equals mono- and disaccharides.

Five of the 18 subjects in the intervention group reached the maximal 15% increase in body weight (four men and one woman). Three of these subjects reached the maximum weight increase ahead of the last study week and subsequently stopped the intervention prematurely. The subject with the steepest increase in body weight was 80 kg at baseline and reached 92 kg (+15%) after 2 weeks. The great majority of the participants complained of epigastric discomfort and profound sense of satiety during the first week, while the remaining weeks in most cases were much more tolerable in this sense.

**Figure 1:**
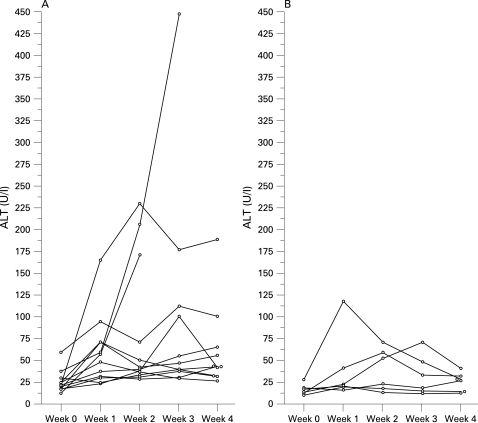
ALT levels at baseline and during the study in men (A, n = 12) and women (B, n = 6) of the intervention group.

All subjects were informed of the test results on a weekly basis, in particular ALT levels. Figure 1 displays the ALT levels during the study in men and women. The one man who reached the highest ALT after just 1 week of hyper-alimentation (164 U/l, fig 1A) was a total abstainer from alcohol. One male participant developed an ALT level of 447 U/l during the third week, and thus was immediately returned to his regular eating habits. However, there were no signs or symptoms of impaired hepatic function (albumin, prothrombin time, alkaline phosphatase, AST and bilirubin were not affected) either in his case or in the other participants that displayed particularly large increases in ALT levels. Furthermore, the ALT elevations subsided either during the study (fig 1) or within a few weeks after the study had finished, in all cases. As shown in table 3 most participants developed pathological ALT levels during the study. Long-term follow-up of ALT after 6 months displayed similar levels as at baseline (baseline 22.4 (12) U/l, follow-up at 6 months 25.5 (22) U/l, p = 0.4 in the paired t test, n = 17, see also table 3).

**Table 3:** Number of subjects in the intervention group displaying serum ALT above reference levels during the study and at follow-up

ALT reference levels	Baseline (men n = 12, women n = 6)	Week 1 (men n = 12, women n = 6)	Week 2 (men n = 12, women n = 6)	Week 3 (men n = 11, women n = 6)	Week 4 (men n = 11*, women n = 6)	Persistent elevation from week 1 to study end	Follow-up after 6 months (n = 17**)
Men >40 U/l†	1	7	5	6	8	5	1
Men >30 U/l††	2	9	11	10	10	8	3
Women >31 U/l†	0	2	3	3	2	1	0
Women >19 U/l††	1	5	4	3	4	3	2

*One subject that had returned to regular eating habits during the fourth week is included.

**One subject lost to follow-up due to studies abroad.

†Upper reference limits for ALT used in NHANES III.^1^

††Updated upper reference limits for ALT.^22^

The increase in ALT was unrelated to the change in waist circumference but tended to correlate with the weight increase (ratio of ALT at study end/baseline ALT to the increase in weight, r = 0.42, p = 0.085). The increase in caloric intake was not correlated with changes in ALT levels (either change from basal level to highest individual ALT, or to ALT change from basal levels to those at study end) or HTGC. The average consumption of fat or proteins during 3 days at the end of the first or third weeks was unrelated to changes in ALT. However, the maximal ALT/baseline ratio correlated with carbohydrate intake during the third week (r = 0.52, p = 0.03, corresponding figures for intake during week 1, r = 0.40, p = 0.1). The intake of sugar (mono- and disaccharides) during week 3 also correlated with the maximal ALT/baseline ALT ratio (intake during the third week, r = 0.62, p = 0.006; corresponding figures for intake at the end of the first week, r = 0.45, p = 0.06), but this correlation was not independent of carbohydrate consumption in a multivariate statistical model (data not shown).

HTGC increased from 1.1 (1.9)% to 2.8 (4.8)% in the intervention group (p = 0.003 by the Wilcoxon signed rank test, table 1). The increase of HTGC did not correlate with the increase in ALT (ratio of ALT at study end/baseline ALT to HTGC at study end/baseline, p = 0.11, Spearman) or intake of fat, carbohydrates and protein. The increase in body weight tended to relate to the increase in HTGC (r = 0.44, p = 0.07) while the increase in the percentage of body fat was clearly related to the increase in HTGC (r = 0.81, p<0.001).

The control group displayed similar anthropometric data as the intervention group but had statistically significantly higher albumin throughout the study month. There was no change in ALT levels in the control group and, as shown in table 1, the baseline ALT was similar in the intervention and the control groups while the difference at the study end between the two groups was statistically significant (p = 0.001).

## DISCUSSION

The most important finding of this study was that during the study period 13 subjects on the high caloric diet developed pathological ALT, according to the updated definition of healthy ranges.^22^ In most subjects this elevation was evident within the first week. Even when the higher levels used in NHANES III^1^ (31 and 40 U/l for women and men, respectively) were considered as upper reference limits for ALT, eight and nine subjects displayed elevated ALT after 1 and 4 weeks of hyper-alimentation, respectively.

All except two subjects in the intervention group had HTGC lower than 5.6% at the end of the study period, thus not fulfilling current criteria for hepatic steatosis.^17^ ^21^ However, a significant increase of HTGC occurred indicating a net retention of lipids within hepatocytes. Thus, similar mechanisms that underlie the pathogenesis of NAFLD may have been induced in the intervention group, and at least partly explain the increased serum ALT levels. Insulin resistance, which is one of the main characteristics of the metabolic syndrome, is the most reproducible factor in the development of NAFLD.^7^ Interestingly, insulin resistance according to homeostasis model assessment (HOMA)^24^ increased significantly in the intervention group during the study period. Moreover, the dietary intervention also induced a visceral accumulation of adipose tissue as shown by the significant increase of waist circumference during the trial. Insulin resistance leads to the accumulation of fat in hepatocytes by two main mechanisms: lipolysis, particularly from visceral adipose tissue, which increases fatty acid concentration in the portal circulation; and hyperinsulinaemia, which leads to increased uptake of fatty acids by the hepatocytes. The increase of fatty acids within hepatocytes may have led to hepatocellular injury, depicted by increased serum ALT in the participating subjects, through multiple mechanisms (for a review see Angulo^18^). It has recently been suggested that hepatocellular injury in NAFLD may be attributable to the combined effects of severe peripheral insulin resistance and relative failure of humoral (adipokine) mediators that combat the effect of high insulin levels on hepatic lipid turnover.^25^ However, ALT levels increased already within a week, a time period after which a significant increase of lipids within hepatocytes seems unlikely to have developed. Furthermore, the magnitude of ALT elevation only tended to correlate with those of HTGC, making it unlikely that fatty infiltration of the liver was the main cause of the rapidly increased ALT levels that were evident in many subjects. Hepatocellular damage due to other reasons than intracellular accumulation of lipids, for example localised inflammation, could potentially explain the elevated ALT levels. Indeed, when examining the relationship of the increase in ALT to intake of different nutrients, fat intake was unrelated to increase in ALT while sugar and carbohydrate intake at week 3 clearly related to the ALT increase. This is in accordance with earlier findings by Solga *et al* who demonstrated that higher carbohydrate intake was significantly associated with an increased risk of biopsy-proven hepatic inflammation in morbidly obese patients undergoing bariatric surgery.^26^ Several participants displayed a spontaneous decrease of ALT levels after the initial increase, during the study, while still gaining weight by the intervention, which is surprising if inflammation was the major mediator of the ALT increase. We believe that a plausible explanation for the ALT elevations in this study may be that the sudden increased supply of metabolic substrates, to the liver, in particular monosaccharides, caused an enzymatic induction in hepatocytes and hence that the normal leakage of ALT through the cell membrane resulted in elevated serum levels. The fact that bilirubin levels decreased in the intervention group is in line with induction of hepatic metabolic capacity rather than frank liver cell damage.

Close clinical follow-up has been suggested as the most cost-effective strategy for asymptomatic patients with negative tests for viral, metabolic and autoimmune markers of liver disease and chronically elevated aminotransferase levels. If the levels are spontaneously normalised or only intermittently elevated, no further evaluation is usually undertaken. However, in many cases high alcohol consumption is suspected even though short-term ingestion of alcohol in healthy individuals has not been shown to be associated with clinical significant increases of aminotransferases.^27^ ^28^ Interestingly, in a study by Belfrage *et al* daily ingestion of 63 g alcohol for 5 weeks induced fatty infiltration of the liver but not elevations of aminotransferases.^29^ In comparison, our study showed that 4 weeks of hyper-alimentation is associated with clinically significant elevations of aminotransferases in many subjects but with development of hepatic steatosis in just one subject out of 18.

The major weakness of our study is that subjects did not undergo liver biopsy to confirm the absence of hepatic pathology at baseline. However, 15 of 18 subjects had normal baseline ALT levels according to the updated definitions^22^ and all subjects except one had HTGC well below the level considered to be abnormal, i.e. 5.6%,^21^ and none had antibodies indicative of viral hepatitis. Thus, underlying liver disease is unlikely among the participating subjects. One subject with an HTGC of 8.7% reported an average weekly alcohol consumption of 340 g, and thus he was considered to have alcoholic steatosis. However, his baseline ALT was also normal (21 U/l) according to the updated definitions. Due to the demanding and unusual nature of the trial it was not possible to recruit enough potential intervention participants to allow randomisation to the intervention or control groups. It should also be noted that the participants were not representative of the general Swedish citizen, as displayed, for example, by the high baseline fibre intake, and of course by inclusion of only lean subjects. Although the levels of serum aminotransferases were the same in controls and in the subjects of the intervention group at baseline, we were not able to analyse this in relation to the diet composition in the controls, since this was not recorded.

Automated routine laboratory testing is frequently part of an annual check-up and physicians are often faced with the problem of a patient with abnormal result on measurement of serum ALT but no symptoms. We conclude that chronically or intermittently elevated ALT can be of purely nutritional origin, particularly when found in the absence of liver steatosis. The fact that 14 out of 18 participants had pathological ALT levels after just 1 week clearly displays that elevated ALT levels after a short over-indulgent holiday can be caused not only by alcohol ingestion, but also by a higher caloric intake than usual combined with a sedentary behaviour. We suggest that in the clinical evaluation of subjects with elevated ALT physicians should include not only questions about alcohol intake, but also explore whether recent excessive food intake has occurred.
